# Decline in telomere length by age and effect modification by gender, allostatic load and comorbidities in National Health and Nutrition Examination Survey (1999-2002)

**DOI:** 10.1371/journal.pone.0221690

**Published:** 2019-08-30

**Authors:** Saruna Ghimire, Carl V. Hill, Francisco S. Sy, Rachelle Rodriguez

**Affiliations:** 1 Department of Sociology and Gerontology, Miami University, Oxford, OH, United States of America; 2 School of Public Health, University of Nevada Las Vegas, Las Vegas, NV, United States of America; 3 Office of Special Populations, National Institute of Aging, Bethesda, MD, United States of America; 4 Purdue Pharma, L.P., Stamford, CT, United States of America; University of California Los Angeles, UNITED STATES

## Abstract

**Background:**

This study aims to assess the decline in telomere length (TL) with age and evaluate effect modification by gender, chronic stress, and comorbidity in a representative sample of the US population.

**Methods:**

Cross-sectional data on 7826 adults with a TL measurement, were included from the National Health and Nutrition Examination Survey, years 1999–2002. The population rate of decline in TL across 10-year age categories was estimated using crude and adjusted regression.

**Results:**

In an adjusted model, the population rate of decline in TL with age was consistent and linear for only three age categories: 20–29 (β = -0.0172, 95% CI: -0.0342, -0.0002), 50–59 (β = -0.0182, 95% CI: -0.0311, -0.0054) and 70–79 (β = -0.0170, 95% CI: -0.0329, -0.0011) years. The population rate of decline in TL with age was significantly greater for males and those with high allostatic load and a history of comorbidities. When the population rate of decline in TL was analyzed by gender in 10-year age bins, a fairly consistent yet statistically non-significant decline for males was observed; however, a trough in the rate was observed for females in the age categories 20–29 years (β = -0.0284, 95% CI: -0.0464, -0.0103) and 50–59 years (β = -0.0211, 95% CI: -0.0391, -0.0032). To further elucidate the gender difference observed in the primary analyses, secondary analyses were conducted with reproductive and hormonal status; a significant inverse association was found between TL and parity, menopause, and age at menopause.

**Conclusions:**

TL was shorter with increasing age and this decline was modified by gender, chronic stress and comorbidities; individuals with chronic morbidity and/or chronic stress and females in their twenties and fifties experienced greater decline. Female reproductive factors, i.e., parity and menopause, were associated with TL.

## Introduction

Telomeres, nucleoprotein structures located at the ends of eukaryotic chromosomes, protect the end of the chromosome from degradation and end-to-end fusion [1]. With each somatic cell division, there is a gradual attrition of the telomere, resulting in telomere length shortening with increasing age [1]. Telomere length (TL) has been proposed as a candidate biomarker of aging [2] whereby longer TL is an indicator of healthy aging. Preservation of TL among healthy individuals, in comparison to those with multiple morbidities, is thought to be one of the several pathways by which the development of chronic diseases and mortality can be explained. Although the association between health status and TL has been fairly well established, it is unclear how quickly TL declines with increasing age, or whether there is any effect modification by gender, chronic stress and morbidities, all of which influence telomere dynamics [3,4,5,6].

Although TL shortens with increasing age [7], TL decreases are not directly proportional with age. Gender, chronic stress, and comorbidities may modify the relationship between age and TL. Females have longer telomeres than males [6]. Chronic psychosocial stress, depression, anxiety, and childhood trauma was associated with shorter TL [4,8,9,10,11]. Perceived stress was associated with lower telomerase activity, and shorter telomere length among healthy premenopausal women [4]. An association between childhood trauma and shorter telomere length in adulthood has been reported [9]. Stressful life events within the last five years was associated with associated with shorter telomeres in Netherlands Study of Depression and Anxiety [12]. A systematic review aimed to examine whether chronic social stress is associated with telomere length throughout the life course, concluded that chronic social stress was associated with shorter telomeres in both early and adult exposures [13]. Further, evidence suggests that shorter telomeres is associated with greater cortisol reactivity to stress, central elements of the physiological stress response system [14,15]. "Allostatic load," also called the wear and tear in the body [16], has been proposed as a conceptualization of cumulative stress exacted on the body through attempts to adapt to life's demands [17]. Stress, an inevitable condition of human existence, has been associated with poor health outcomes [16]. Limited research has looked at the relationship between TL and allostatic load [3].

TL has been linked to various morbidities such as diabetes [18], heart disease [19,20,21,22], hypertension [23] cancer [24], and depression [25], as well as overall mortality [26,27]. A meta-analysis of 62 population based studies found a non-significant association between short telomeres and overall risk of cancer but an increased risk for gastrointestinal tumor and head and neck cancer indicating that telomeres may play diverse roles for risk in different cancers [24]. In contrast, a Mendelian Randomization Study reported an increased risk for several cancers with longer telomeres but reduced risk for cardiovascular diseases [28]. Another meta-analysis reported negative association between depression and TL [25]. The Charlson comorbidity index (CCI) is commonly used to provide a cumulative weighted score of 17 comorbid conditions [29]. The measure of decline in TL with age by allostatic load and comorbidities could be useful particularly in elucidating the biologic pathway by which chronic stress and comorbidities can affect TL and accelerate the rate of aging. Once this pathway is better understood, this information can be used to develop effective prevention measures in at-risk populations. Therefore, we aim to assess the decline in TL with age and evaluate any effect modification by gender, chronic stress and comorbidities in a representative sample of the US population. Guided by the results of the primary analyses and in order to further elucidate the observed gender differences, secondary analyses were conducted to examine the association between TL, parity and menopause.

## Methods

### Study design

National Health and Nutrition Examination Survey (NHANES) is a cross-sectional nationally representative survey of the US civilian noninstitutionalized population, conducted using complex, multistage, stratified, clustered sampling [30,31]. Details of NHANES methodology have been reported elsewhere [30,31]. We used data from the cycles 1999–2000 and 2001–2002; combining them following the National Center for Health Statistics (NCHS) recommendations [31].

### Study participants

NHANES 1999–2000 and 2001–2002 included a total of 9965 and 11,039 participants, respectively. For our analyses, we included those aged ≥ 20 years who had a measure of telomere length, for a total of 7826 individuals from both the cycles, NHANES 1999–2002.

### Ethical approval

NHANES was approved by the NCHS Research Ethics Review Board (https://www.cdc.gov/nchs/nhanes/irba98.htm). All participants provided written informed consent. The Institutional Review Board at the University of Nevada Las Vegas approved the current study.

### Measurements

#### Telomere length

Telomere length in leukocytes was measured from whole blood using the quantitative polymerase chain reaction method, described in detail elsewhere [32,33]. The Mean T/S ratio, which is the measure of TL relative to standard reference DNA, were provided in the NHANES dataset. During data analysis, the T/S ratio was converted to kilobase pairs (kbp) using the following formula: (3,274 + 2,413 * (T/S))/1000.

#### Allostatic load

Chronic stress, a hypothesized effect modifier, was measured in terms of allostatic load (AL), quantified using nine biomarkers of cardiovascular, inflammatory, and metabolic system functioning. The nine biomarkers with corresponding cutoffs [34,35] were: systolic blood pressure ≥ 140 mm Hg, diastolic blood pressure ≥ 90 mm Hg, heart rate ≥ 90 beats/minute, total cholesterol level ≥ 240 mg/dL, high-density lipoprotein (HDL) cholesterol < 40mg/dL, BMI ≥ 30 kg/m2, glycosylated hemoglobin ≥ 6.4%, C-reactive protein ≥ 0.3 mg/dL, and albumin < 3.8 g/dL. Each measure was coded as a dichotomous variable at the cutoff (1, if the respondent had indicated the condition; 0, if otherwise). The cumulative score of the nine indicators was then converted into a dichotomous variable, with high AL defined as an AL score ≥ 3. The same cutoff values and measures have been used to quantify allostatic load with the NHANES dataset in previous studies [34,35].

#### Comorbidity

We calculated the Charlson Comorbidity Index [29], another hypothesized effect modifier, to account for the impact of any comorbid conditions on telomere length. Different health conditions included in calculating CCI, their definations and corresponding weights in the calculation, are provided in S1 Table. Because a score of ≥4 points is associated with an estimated 53% 10-year mortality, a weighted combined index score of ≥4 points was used to define a history of chronic comorbid conditions [29].

#### Other covariates

Covariates for this study, selected based on the literature, were race/ethnicity, educational attainment, socioeconomic status (SES), and physical activity levels [6,33,36].

Age (in years and 10-year categories), gender, race/ethnicity (nominal: Hispanic, NH white, NH Black, and others including multi-racial); educational level (ordinal: <12th grade, high school graduate/some college, and college graduate or above); and marital status (nominal: married/living with partner, divorced/widowed/separated, and never married) were self-reported by the participants. SES was measured on a continuous scale, in terms of poverty income ratio (PIR). PIR, the ratio of family income to the poverty threshold, calculated following the U.S. Department of Health and Human Services’ poverty guidelines and described in detail elsewhere [31]. BMI, the ratio of weight/height^2^ measured in kg/m^2^, was available as a continuous measure and was categorized as normal weight (<25 kg/m^2^), overweight (25- <30 kg/m^2^) and obese (≥30 kg/m^2^). Physical activity was defined as participants’ self-reported participation in at least 10 minutes of moderate or vigorous activity or muscle strengthening activities in the previous 30 days [33]. Following the definition for menopause proposed by McKinlay [37], menopause was defined as one or more of the given criteria: over 55 years, had a hysterectomy or both ovaries removed, and menopause as the reason for no periods in the past 12 months. Age at menopause as well as number of pregnancies resulting in live birth were self-reported by women.

### Statistical analyses

Sample weights were adjusted according to NHANES guidelines to generate a nationally representative sample [31]. For nominal variables, percentages with 95% CIs are provided; for continuous variables, unadjusted means with standard error (SEM) are provided (Table 1). TL was divided into quartiles based on the weighted population distribution. Covariate characteristics, between the quartiles of TL, were compared using Rao-Scott Chi-Square tests and analysis of variance. Linear regression with TL (kbp) as the outcome was used to assess the association with socio-demographic factors, biomarkers of allostatic load, and reproductive factors among females. Initially, univariate models were evaluated, then a group of covariates (age, gender, race/ethnicity, education, PIR, and physical activity) were added to the model.

**Table 1:** Socio-demographic characteristics of the study participants by quartiles of leukocyte telomere length- NHANES 1999–2002.

	Overall (N = 7826)		Quartiles, % (95% CI)				
	N	% (95% CI)	One (N = 2415)	Two (N = 2120)	Three (N = 1633)	Four (N = 1658)	p-Value
			Range: 4.2-<5.4 kbps	Range:5.4-<5.8 kbps	Range: 5.8-<6.2 kbps	Range: ≥6.2 kbps	
Age, years, mean ±SEM	7826	45.2 ±0.4	54.6 ±0.7	46.2 ±0.8	41.5 ±0.7	37.4 ±0.8	**<0.001** ^a^
LTL, kbp, mean ±SEM^b^	7826	5.8 ± 0.04	5.1 ±0.0	5.6 ±0.0	6.0 ±0.0	6.7 ±0.0	**<0.001** ^a^
Gender							
*Male*	3770	48.6 (47.6–49.5)	50.7 (48.3–53.2)	48.0 (45.1–50.9)	47.7 (45.5–49.9)	47.7 (45.2–50.3)	0.287
*Female*	4056	51.4 (50.5–52.4)	49.3 (46.8–51.7)	52.0 (49.1–54.9)	52.3 (50.1–54.5)	52.3 (49.7–54.8)	
Race/Ethnicity							
*Hispanic*	2292	13.8 (9.7–17.8)	11.8 (6.0–17.7)	13.4 (9.3–17.4)	14.2 (9.7–18.6)	15.9 (11.1–20.6)	**<0.001**
*NH White*	3965	72.8 (69.0–76.5)	77.4 (71.8–82.9)	74.7 (70.5–78.9)	71.1 (66.4–75.8)	67.2 (62.4–72.0)	
*NH Black*	1333	9.4 (7.2–11.6)	7.2 (5.2–9.2)	8.1 (5.6–10.6)	9.8 (7.6–12.1)	12.8 (9.4–16.3)	
*Other*	236	4.1 (2.7–5.4)	3.6 (1.8–5.5)	3.9 (2.7–5.0)	4.9 (3.1–6.7)	4.0 (1.8–6.3)	
Educational Status							
*<12th Grade*	2640	21.3 (19.4–23.2)	26.3 (23.4–29.2)	20.6 (18.3–22.9)	20.8 (17.4–24.2)	17.1 (15.3–18.9)	**<0.001**
*High School/Some College*	3733	54.6 (51.7–57.5)	53.0 (49.1–56.9)	55.2 (51.6–58.8)	52.1 (47.1–57.1)	57.9 (53.4–62.5)	
*College Graduate*	1441	24.1 (20.7–27.5)	20.7 (16.8–24.6)	24.2 (20.7–27.7)	27.1 (21.6–32.7)	24.9 (19.8–30.1)	
Marital Status							
*Married/Living with Partner*	4759	65.1 (63.0–67.2)	68.8 (66.4–71.1)	67.3 (64.2–70.3)	63.9 (60.5–67.3)	59.5 (55.7–63.3)	**<0.001**
*Divorced/Widowed/Separated*	1566	17.5 (16.2–18.9)	23.0 (21.0–24.9)	19.4 (17.3–21.6)	14.4 (11.9–16.8)	12.1 (9.8–14.4)	
*Never Married*	1123	17.4 (15.6–19.1)	8.3 (6.8–9.8)	13.3 (10.8–15.8)	21.8 (19.1–24.4)	28.4 (25.4–31.4)	
Family PIR, mean ±SEM	7128	3.0 ±0.1	3.0 ±0.1	3.1 ±0.1	3.1 ±0.1	2.8 ±0.1	**<0.001** ^a^
BMI, kg/m^2^, mean ±SEM	7577	28.0 ±0.1	28.7 ±0.2	28.2 ±0.2	27.8 ±0.2	27.4 ±0.3	**<0.001** ^a^
Physical Activity							**<0.001**
*Physically active*	4552	66.3 (63.5–69.1)	59.4 (55.7–63.2)	66.6 (62.8–70.4)	69.2 (66.6–71.8)	70.7 (66.5–74.8)	
*Physically inactive*	3271	33.7 (30.9–36.5)	40.6 (36.8–44.3)	33.4 (29.6–37.2)	30.8 (28.2–33.4)	29.3 (25.2–33.5)	
Allostatic load, mean± SEM ^b^	7826	2.4 ±0.0	2.7 ±0.0	2.5 ±0.0	2.4 ±0.0	2.2 ±0.1	**<0.001** ^a^
CCI, mean ±SEM ^b^	7826	1.5 ±0.0	2.2 ±0.1	1.5 ±0.1	1.2 ±0.1	1.0 ±0.1	**<0.001** ^a^

^a^: p-values from one-way ANOVA; all others from a Chi-square test.

^b^ SEM values of 0 indicates a value <0.1. Abbreviations: BMI: body mass index, CCI: Charleston’s comorbidity index, CI: confidence interval, Kbp: kilo base pairs, LTL: leucocyte telomere length, NH: Non-Hispanic, PIR: poverty income ratio, SEM: standard error of mean. Values expressed are % (95% CI) unless otherwise stated.

The main outcome of this study is rate of decline in TL, which would best use a longitudinal design study. Since this is a cross-sectional study, we aim to examine the cross-sectional rates of TL decline as a proxy for rate of decline in TL by age. For this we are assuming that an individual with a TL at a certain age will, later in life, have the same telomere length as another individual at an older age with similar covariates. Therefore, the rate we are measuring is a population rate of decline in TL, not an individual rate of decline. To measure the population rate of decline in TL in different age categories, we used 10-year bins and calculated the slope of the rate of decline in TL as a function of age in each bin, using a linear regression with TL (kbp) as outcome and age (years) as predictor. All population rates of decline in TL are adjusted for gender, ethnicity, physical activity, CCI, and allostatic load. A two-tailed p-value less than 0.05 was considered statistically significant. Data analyses were performed using the survey procedures that account for the weights and complex survey design of NHANES, in SAS 9.4 (SAS Institute Inc., Cary, NC).

## Results

### Participant’s characteristics

The mean (±SE) age and TL of the participants were 45.2±0.4 years and 5.8±0.04 kbp, respectively (Table 1). Participants with shorter TL, i.e., in lower quartiles, were older, NH White, married, and had higher BMI, allostatic load, and CCI. A gradual decreasing trend in mean age, BMI, allostatic load and CCI was noted from lowest to highest quartiles (Table 1).

### Telomere length and associated factors

In analyses adjusted for age, gender, race/ethnicity, education, PIR, and physical activity (Table 2), increasing age, and higher heart rate, BMI, and C-reactive protein were associated with lower TL. Likewise, NH Black compared to NH White and unmarried compared to married/living with a partner had higher TL. A further stratified analysis was conducted to assess the role of age and gender in the association between marital status and TL. We found that the marital status and TL association was significant only for younger (20–29 years) females (β: 0.5194, 95%CI: 0.1129–0.9258, p-value: 0.0141) and older (80 years and above) females (β: 0.2770, 95%CI: 0.1217–0.4323, p-value: 0.001). Although female gender demonstrated a positive trend and family PIR demonstrated an inverse trend with TL, the findings did not reach the statistical significance of <0.05 (Table 2).

**Table 2:** Multivariable regression for factors associated with telomere length- NHANES 1999–2002.

	Model 1 unadjusted			Model 2 adjusted ^a^		
	β	95%CI	p-value	β	95%CI	p-value
Age	**-0.0146**	**-0.0162, -0.0130**	**<0.001**	**-0.0142**	**-0.0157, -0.0127**	**<0.001**
Gender (Reference = Male)						
*Female*	0.0252	-0.0265, 0.0769	0.328	0.0460	-0.0015, 0.0934	0.0569
Race/Ethnicity (Reference = NH White)						
*Hispanic*	0.0681	-0.0692, 0.2054	0.319	-0.0053	-0.137, 0.1264	0.9352
*NH Black*	**0.1687**	**0.0802, 0.2572**	**<0.001**	**0.1186**	**0.028, 0.2092**	**0.0121**
*Other*	0.0396	-0.1005, 0.1797	0.568	-0.0141	-0.1502, 0.122	0.8334
Educational Status (Reference = High School)						
*<12th Grade*	**-0.1203**	**-0.1825, -0.0581**	**<0.001**	-0.0679	-0.1365, 0.0006	0.052
*College Graduate*	-0.0023	-0.0689, 0.0642	0.943	0.0305	-0.0357, 0.0967	0.354
Marital Status (Reference = Married/with Partner)						
*Divorced/Widowed/Separated*	**-0.1111**	**-0.1655, -0.0566**	**<0.001**	-0.0128	-0.0722, 0.0465	0.6614
*Never Married*	**0.3162**	**0.2422, 0.3901**	**<0.001**	**0.0979**	**0.0233, 0.1726**	**0.0119**
Family PIR	-0.0127	-0.0354, 0.0100	0.261	-0.0048	-0.0275, 0.018	0.6699
Physical Activity (Reference = Physically Active)						
Physically Inactive	**-0.1158**	**-0.1653, -0.0663**	**<0.001**	-0.0151	-0.0537, 0.0236	0.4316
Biomarkers of allostatic load						
*Systolic blood pressure*	**-0.0030**	**-0.0044, -0.0015**	**<0.001**	0.0011	-0.0001, 0.0023	0.0631
*Diastolic blood pressure*	**0.0041**	**0.0022, 0.0060**	**<0.001**	0.0001	-0.0018, 0.0018	0.9755
*Heart rate*	-0.0008	-0.0026, 0.0009	0.325	**-0.0021**	**-0.0036, -0.0006**	**0.0074**
*Total cholesterol*	-0.0002	-0.0007, 0.0004	0.516	-0.0001	-0.0007, 0.0004	0.5865
*High-density lipoprotein cholesterol*	**0.0027**	**0.0009, 0.0044**	**0.004**	**0.0023**	**0.0005, 0.0041**	**0.0134**
*BMI (kg/m2)*	**-0.0093**	**-0.0133, -0.0052**	**<0.001**	**-0.0068**	**-0.0105, -0.0031**	**0.0007**
*Glycosylated hemoglobin*	**-0.0370**	**-0.0573, -0.0167**	**<0.001**	-0.0004	-0.0257, 0.0249	0.9751
*C-reactive protein*	**-0.0538**	**-0.0993, -0.0083**	**0.022**	**-0.0558**	**-0.0903, -0.0214**	**0.0025**
*Albumin*	0.1664	-0.3897, 0.7225	0.545	0.2317	-0.6846, 1.1481	0.609
Allostatic load (Reference = Low)						
*High*	**-0.1735**	**-0.2226, -0.1244**	**<0.001**	**-0.0697**	**-0.1166, -0.0228**	**0.005**
Charleston Comorbidity Index	**-0.0723**	**-0.0823, -0.0624**	**<0.001**	-0.0044	-0.0173, 0.0086	0.4971

DV: Telomere length in kbp

^**a**^ Adjusted for age, gender, race/ethnicity, education, PIR, and physical activity. Abbreviations: BMI: body mass index, CI: confidence interval, NH: Non-Hispanic, PIR: poverty income ratio. p-value less than 0.05 are bold.

Of the nine biomarkers of allostatic load, after controlling for covariates, only the regression coefficients of heart rate, HDL cholesterol, BMI, and C-reactive protein had the 95% CIs that did not include the null of no association (Table 2); while a higher heart rate, BMI, and C-reactive protein was associated with shorter TL, a higher value of HDL cholesterol preserved it. In an unadjusted analysis, a one-unit difference in allostatic load and CCI were associated with 17.3% and 7.2% decrease in TL, respectively. However, the estimates for CCI did not remain statistically significant when controlled for covariates. Specifically, age had a strong confounding effect on our estimates because most of the covariates with a significant coefficient in unadjusted models retained their statistical significance when adjusted for other covariates except age (S2 Table).

### Cross-sectional population rates of telomere length decline

The population rates of decline in TL stratified by 10-year age bins showed that decline in TL with age was initiated early in life (20–29 years) and was consistent and linear for only three age categories: 20–29 (β = -0.0172, 95% CI: -0.0342, -0.0002), 50–59 (β = -0.0182, 95% CI: -0.0311, -0.0054) and 70–79 (β = -0.0170, 95% CI: -0.0329, -0.0011) years. The population rate of decline in TL was sharp in the age category 70–79 years; whereas, in the oldest age category examined (80–89 years), the rate of decline (β = -0.0096, 95% CI: -0.0412, 0.0220) was highly variable and did not follow a linear pattern. Likewise, the population rate of decline in TL stratified by allostatic load and comorbid conditions showed a significantly greater rate of decline among those with high allostatic load (β = -0.0122, 95% CI: -0.0143, -0.0101), and history of comorbid conditions (β = -0.0133, 95% CI: -0.0164, -0.0102).

**Fig 1:**
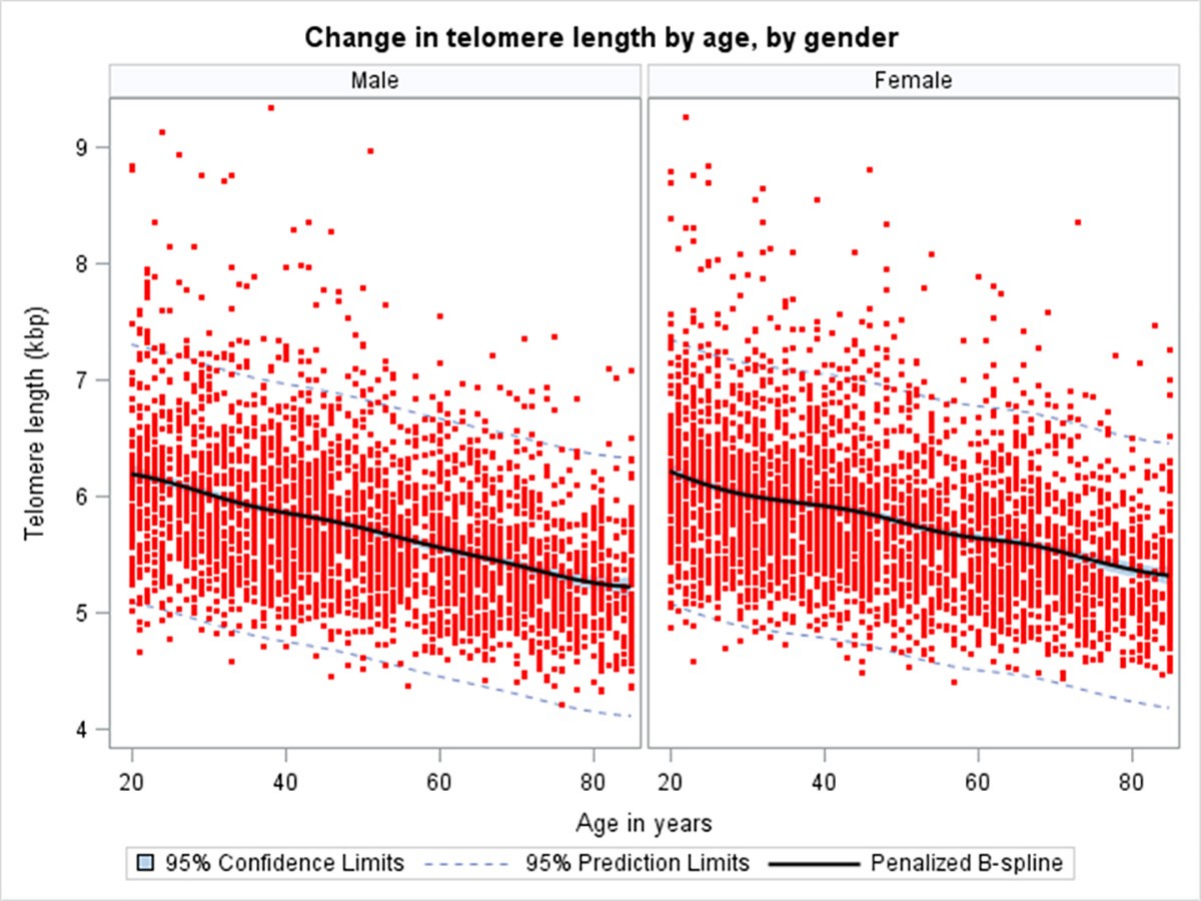
Telomere Length by Age and Gender in NHANES 1999–2002. **(a) Male** (β = -0.0153; p-value <0.001), **(b) Female** (β = -0.0129; p-value <0.001). Estimates, obtained from linear regression with telomere length (kbps) as outcome and age (years) as predictor, are adjusted for ethnicity, physical activity, allostatic load and comorbidity index.

Gender stratified analyses, adjusted for ethnicity, physical activity, CCI, and allostatic load, (Fig 1) showed a significantly greater rate of decline for males (β = -0.0153, 95% CI: -0.0171, -0.0135) compared to females (β = -0.0128, 95% CI: -0.0153, -0.0103). When the population rate of decline in TL was further stratified by gender in 10-year age bin (Table 3), we observed a fairly consistent population rate of decline in TL for male; none of the regression coefficients were statistically significant (Table 3). Interestingly, for females, a peak in the population rate of decline in TL was observed in the age categories of 20–29 years (β = -0.0284, 95% CI: -0.0464, -0.0103) and 50–59 years (β = -0.0211, 95% CI: -0.0391, -0.0032). Although women in the age categories of 30–39 years and 60–69 years displayed a minimal reduction in TL, the estimates were not statistically significant (Table 3).

**Table 3:** Decline in telomere length by gender, across age groups, NHANES 1999–2002.

Age group, years	Male			Female		
	β	95% CI	p-value	β	95% CI	p-value
20–29	-0.0080	-0.0367, 0.0208	0.5763	**-0.0284**	**-0.0468, -0.0099**	**0.0038**
30–39	-0.0078	-0.0290, 0.0135	0.4621	-0.0012	-0.0214, 0.0191	0.9084
40–49	-0.0060	-0.0290, 0.0170	0.5985	-0.0130	-0.0321, 0.0060	0.1724
50–59	-0.0166	-0.0337, 0.0005	0.0571	**-0.0216**	**-0.0396, -0.0036**	**0.0202**
60–69	-0.0124	-0.0333, 0.0085	0.2342	0.0040	-0.0179, 0.0258	0.7134
70–79	-0.0215	-0.0439, 0.0010	0.0599	-0.0146	-0.0326, 0.0034	0.1079
80 and above	-0.0116	-0.0377, 0.0145	0.3700	-0.0075	-0.0519, 0.0369	0.7334

Estimates are slope from a linear regression with telomere length (kbps) as outcome and age (years) as predictor, by age category and gender, adjusted for ethnicity, allostatic load, and comorbidity index. p-value less than 0.05 are bold.

To explore the heterogeneity in the population rate of decline in TL among females, we conducted additional analyses for women’s reproductive history, particularly parity and menopause, since ages 20–29 and 50–59 years are common ages for parity and menopause, respectively. After controlling for ethnicity, education, PIR, and BMI, the number of live children a woman had was negatively associated with TL (β = -0.0318, 95% CI: -0.0510, -0.0125). Further, compared to nulliparous women, decline in TL was noted for women with live births (S1 Fig). Similarly, women’s menopausal status (β = -0.2875, 95% CI: -0.3517, -0.2232), age at menopause (β = -0.0077, 95% CI: -0.0130, -0.0023) as well as years passed since menopause (β = -0.0095, 95% CI: -0.0127, -0.0063) were inversely associated with TL. Further, variation in slope of decline in TL with years passed since menopause was noted by women’s weight status (S2 Fig). However, these findings were no longer statistically significant once we controlled for age (S3 Table).

## Discussion

Overall, TL significantly reduced with age, but this decline was modified by gender, chronic stress and comorbidities; those with chronic morbidities and chronic stress experienced greater decline. Gender stratified analysis was more interesting, showing a fairly consistent and statistically non-significant rate for male but a substantial trough in TL for females in the age categories 20–29 and 50–59 years. Female reproductive factors, i.e., parity and menopause, were associated with TL.

Decline in TL with age is unanimously supported by the literature [33,38]. Heterogeneity in rate of decline in TL is suggested by a previous study from the Erasmus Rucphen Family (ERF) data, which found a significant reduction in variance in TL from young adulthood to old age by using the TL of grandchildren as a proxy for participants’ TL at childhood [38]. For females, a trough in the adjusted population rate of decline in TL was noted in early (20–29) and midlife (50–59) years. In general, for females the 20–29 and 50–59 age bands are periods of important hormonal changes, as these are the common ages for parity and menopause, respectively. Consistent with previous studies, we found a significant negative association between TL and parity, menopausal status, and age at menopause [39]. The observed inverse relationship between TL and parity is supported by the life history theory, which postulates that the energy used during reproduction reduces the energy available for tissue maintenance given that the amount of energy an organism can mobilize at any given time is finite, and that poor tissue maintenance, in turn, leads to faster cellular degradation and aging [40]. Biologically, estrogen deficiency or over-activity may cause either ovarian tissue aging or tumorigenesis, respectively, through estrogen regulation of telomere remodeling [41]. Therefore, telomere attrition rate should accelerate after menopause in response to a decrease in estrogen [41]. In vitro, telomerase activity is upregulated by estrogen [42,43]. Population-based studies also provide evidence that greater estrogen exposure, as measured by the use of hormone therapy [44], and longer duration of reproductive years [45], are related to significantly higher TL in postmenopausal women. Oxidative stress and proinflammatory cytokines have also been associated with telomere shortening [46,47]; thus, antioxidants aid in the attenuation of telomere shortening [48,49]. Estrogen is known to have antioxidant properties [50,51]; thus, the conferred protective role may be due to its ability to lower oxidative stress and reduce inflammation [52,53].

This study found that one-unit increases in allostatic load were associated with 7% decreases in TL after controlling for covariates. Further analyses stratified by allostatic load levels showed a greater decline in TL among those with higher allostatic load. The allostatic load model posits that repeated or inadequate physiological adaption to social and environmental stress over time results in dysregulation of cortisol (via dysfunction of the hypothalamic-pituitary-adrenal axis) and catecholamines (via the sympathetic nervous system), which may in turn result in dysfunction of the body’s cardiovascular, immune, and metabolic systems [16]. Therefore, it is likely that TL may serve as an important cellular-based indicator of systemic allostatic load.

The associations between TL and various morbidities such as diabetes [18], heart disease [19,20,21,22], hypertension [23] cancer [24], and depression [25], as well as overall mortality [26,27] have been established. In this study, one-unit increases in comorbidities, as measured by the Charlson Comorbidity Index, was associated with 7.2% decreases in TL, although findings lost significance after adjusting for age. Regardless, there is biological plausibility linking comorbidities with TL. Additionally, since TL is strongly correlated with chronological age, the latter had a powerful confounding effect on most of our estimates which lost statistical significance when adjusted for age. Further, analyses stratified by comorbidity levels showed a greater decline in TL among those with four or more comorbidities. It has been suggested that telomere shortening might contribute to various morbidities through pathways involving cellular senescence, chronic inflammation and endothelial dysfunction [54,55].

Many of the covariates we selected were associated with TL. TL in our study was positively correlated with HDL cholesterol and inversely correlated with BMI and heart rate [36]. The existing literature on race/ethnicity and TL is inconsistent [33,56]. In our study, NH Black had higher allostatic load compared to NH White (OR = 1.32, 95% CI = 1.13–1.53), after adjustment for age, gender, and PIR. Despite the higher allostatic load, in overall and stratified analysis by allostatic load status, NH Black had higher TL than NH White. Although unexpected given that African-Americans experience greater stress in various life domains, sociocultural factors such as social support and religion/spirituality may enhance resilience when dealing with psychological distress for this group [57]. These factors may nurture coping efficacy which in turn fosters an ability to manage adversity [57]. More research is needed to understand relationships between high effort coping styles and TL for racial and ethnic populations in the United States. Another surprising finding in our study was never having been married was associated with longer TL than being married. This finding conflicts with other studies that showed being married is associated with longer TL [58,59]. In general, being unmarried is associated with poor health outcomes, presence of systemic inflammation [60,61] increased mortality risk, and a shorter lifespan [60,61,62,63]. Although the protective role of marriage is not completely understood, it has been hypothesized that the social, emotional, and financial support provided by a spouse/partner acts as a buffer to life stressors [64]. Even among laboratory animals, social isolation was related to increased oxidative stress [65] which in turn is related to telomere attrition. In our stratified analyses, we found that age and gender played a role in the association between marital status and TL. Thus, different mediators and moderators may explain the inconsistency observed in the relationship between marital status and TL attrition, which should be explored in future research.

### Strengths, limitations and implications

The strengths of the study include a large sample size, a nationally representative sample of non-institutionalized American adults, rigorous methodology and the comprehensive quality control procedures of NHANES. The statistical analyses have been adjusted for study weights and complex survey design to reduce errors in estimation. The cross-sectional design of the study is the primary limitation, and no causation should be inferred from this study. Limitations were also observed for the quantification of allostatic load and CCI. Currently no gold standard measure exists to quantify allostatic load, and while our approach was similar to others [34,35], the extent to which the measures of allostatic load actually reflect the complex concept of “wear and tear” is uncertain, which may impact the accuracy of the measurement, and consequently, the quality of the evidence generated. Of the 17 comorbidities used in the original CCI, we were unable to include the measures of hemiplegia and metastatic cancer due to unavailability of data on these conditions in NHANES 1999–2002. Some of the disease statuses used in calculating CCI were self-reported. Lastly, the possibility of residual confounding due to unmeasured covariates cannot be ruled out.

If the findings from this current study could be replicated with longitudinal data, there may be several important implications. First, our findings imply that interventions aimed at preserving TL should be targeted at younger ages, not just at old age. The heterogeneity in decline in TL and absence of a linear pattern in the oldest age category (≥80 years) may also partially explain the lack of statistical association between TL and survival, as seen in some studies conducted among the elderly. For women, a trough in decline in TL with age was noted in early and midlife years. Although 20–29 and 50–59 years are common ages for first childbirth and menopause, respectively, the underlying cause of the decline in these age categories is still not clear and should be addressed by future research.

## Conclusions

We found shorter TL with increasing age; this decline was modified by gender, chronic stress and comorbidities. Females in their twenties and fifties and those with chronic morbidities and/or chronic stress experienced greater TL decline. Female reproductive factors, i.e., parity and menopause, were associated with TL. Given the cross-sectional design of our study, future research should attempt to replicate our findings, specifically those related to parity and menopause, in a longitudinal design. Females in their twenties and fifties are potential subgroups of interest for any interventions or programs aimed at preserving TL.

## Supporting information

S1 Fig Association between telomere length and parity in NHANES 1999–2002. Y-axis represents the slope of change in telomere length with the number of live births. Regression coefficients adjusted for ethnicity, education, poverty income ratio, and body mass index.
(TIF)

S2 Fig Association between telomere length and years passed since menopause, by BMI status of women in NHANES 1999–2002. The slope for each weight category is: Normal weight (β = -0.0101; p = 0.001), Overweight (β = -0.0111; p = 0.005) and Obese (β = -0.0076; p = 0.007). Estimates adjusted for ethnicity, education, poverty income ratio, and body mass index.
(TIF)

S1 Table Comorbid conditions included in Charlson’s comorbidity index. (DOCX)

S2 Table Multivariable regression for factors associated with telomere length- NHANES 1999–2002. (DOCX)

S3 Table Multivariable regression for women’s reproductive history associated with telomere length- NHANES 1999–2002. (DOCX)

## References

[1] Blackburn EH, Greider CW, Szostak JW (2006) Telomeres and telomerase: the path from maize, Tetrahymena and yeast to human cancer and aging. Nat Med 12: 1133–1138. 10.1038/nm1006-1133 17024208

[2] von Zglinicki T, Martin-Ruiz CM (2005) Telomeres as biomarkers for ageing and age-related diseases. Curr Mol Med 5: 197–203. 15974873 10.2174/1566524053586545

[3] Ahrens KA, Rossen LM, Simon AE (2016) Relationship Between Mean Leucocyte Telomere Length and Measures of Allostatic Load in US Reproductive-Aged Women, NHANES 1999–2002. Paediatr Perinat Epidemiol 30: 325–335. 10.1111/ppe.12277 26854139 PMC6697084

[4] Epel ES, Blackburn EH, Lin J, Dhabhar FS, Adler NE, Morrow JD, et al (2004) Accelerated telomere shortening in response to life stress. Proc Natl Acad Sci U S A 101: 17312–17315. 10.1073/pnas.0407162101 15574496 PMC534658

[5] Fuster JJ, Andres V (2006) Telomere biology and cardiovascular disease. Circ Res 99: 1167–1180. 10.1161/01.RES.0000251281.00845.18 17122447

[6] Gardner M, Bann D, Wiley L, Cooper R, Hardy R, Nitsch D, et al (2014) Gender and telomere length: systematic review and meta-analysis. Exp Gerontol 51: 15–27. 10.1016/j.exger.2013.12.004 24365661 PMC4523138

[7] Slagboom PE, Droog S, Boomsma DI (1994) Genetic determination of telomere size in humans: a twin study of three age groups. Am J Hum Genet 55: 876–882. 7977349 PMC1918314

[8] Okereke OI, Prescott J, Wong JY, Han J, Rexrode KM, De Vivo I. (2012) High phobic anxiety is related to lower leukocyte telomere length in women. PLoS One 7: e40516 10.1371/journal.pone.0040516 22808180 PMC3394740

[9] Tyrka AR, Price LH, Kao HT, Porton B, Marsella SA, Carpenter LL. (2010) Childhood maltreatment and telomere shortening: preliminary support for an effect of early stress on cellular aging. Biol Psychiatry 67: 531–534. 10.1016/j.biopsych.2009.08.014 19828140 PMC2853238

[10] Simon NM, Smoller JW, McNamara KL, Maser RS, Zalta AK, Pollack MH, et al (2006) Telomere shortening and mood disorders: preliminary support for a chronic stress model of accelerated aging. Biol Psychiatry 60: 432–435. 10.1016/j.biopsych.2006.02.004 16581033

[11] O'Donovan A, Epel E, Lin J, Wolkowitz O, Cohen B, Maguen S, et al (2011) Childhood trauma associated with short leukocyte telomere length in posttraumatic stress disorder. Biol Psychiatry 70: 465–471. 10.1016/j.biopsych.2011.01.035 21489410 PMC3152637

[12] Verhoeven JE, van Oppen P, Puterman E, Elzinga B, Penninx BW (2015) The Association of Early and Recent Psychosocial Life Stress With Leukocyte Telomere Length. Psychosom Med 77: 882–891. 10.1097/PSY.0000000000000226 26374947

[13] Oliveira BS, Zunzunegui MV, Quinlan J, Fahmi H, Tu MT, Guerra RO. (2016) Systematic review of the association between chronic social stress and telomere length: A life course perspective. Ageing Res Rev 26: 37–52. 10.1016/j.arr.2015.12.006 26732034

[14] Gotlib IH, LeMoult J, Colich NL, Foland-Ross LC, Hallmayer J, Joormann J, et al (2015) Telomere length and cortisol reactivity in children of depressed mothers. Mol Psychiatry 20: 615–620. 10.1038/mp.2014.119 25266121 PMC4419149

[15] Tomiyama AJ, O'Donovan A, Lin J, Puterman E, Lazaro A, Chan J, et al (2012) Does cellular aging relate to patterns of allostasis? An examination of basal and stress reactive HPA axis activity and telomere length. Physiol Behav 106: 40–45. 10.1016/j.physbeh.2011.11.016 22138440 PMC3361080

[16] McEwen BS, Seeman T (1999) Protective and damaging effects of mediators of stress. Elaborating and testing the concepts of allostasis and allostatic load. Ann N Y Acad Sci 896: 30–47. 10.1111/j.1749-6632.1999.tb08103.x 10681886

[17] Seeman TE, McEwen BS, Rowe JW, Singer BH (2001) Allostatic load as a marker of cumulative biological risk: MacArthur studies of successful aging. Proc Natl Acad Sci U S A 98: 4770–4775. 10.1073/pnas.081072698 11287659 PMC31909

[18] Sampson MJ, Winterbone MS, Hughes JC, Dozio N, Hughes DA (2006) Monocyte telomere shortening and oxidative DNA damage in type 2 diabetes. Diabetes Care 29: 283–289. 10.2337/diacare.29.02.06.dc05-1715 16443874

[19] Haycock PC, Heydon EE, Kaptoge S, Butterworth AS, Thompson A, Willeit P. (2014) Leucocyte telomere length and risk of cardiovascular disease: systematic review and meta-analysis. BMJ 349: g4227 10.1136/bmj.g4227 25006006 PMC4086028

[20] Brouilette SW, Moore JS, McMahon AD, Thompson JR, Ford I, Shepherd J, et al (2007) Telomere length, risk of coronary heart disease, and statin treatment in the West of Scotland Primary Prevention Study: a nested case-control study. Lancet 369: 107–114. 10.1016/S0140-6736(07)60071-3 17223473

[21] van der Harst P, van der Steege G, de Boer RA, Voors AA, Hall AS, Mulder MJ, et al (2007) Telomere length of circulating leukocytes is decreased in patients with chronic heart failure. J Am Coll Cardiol 49: 1459–1464. 10.1016/j.jacc.2007.01.027 17397675

[22] Weischer M, Bojesen SE, Cawthon RM, Freiberg JJ, Tybjaerg-Hansen A, Nordestgaard BG. (2012) Short telomere length, myocardial infarction, ischemic heart disease, and early death. Arterioscler Thromb Vasc Biol 32: 822–829. 10.1161/ATVBAHA.111.237271 22199369

[23] Yang Z, Huang X, Jiang H, Zhang Y, Liu H, Qin C, et al (2009) Short telomeres and prognosis of hypertension in a chinese population. Hypertension 53: 639–645. 10.1161/HYPERTENSIONAHA.108.123752 19255364 PMC2890281

[24] Zhu X, Han W, Xue W, Zou Y, Xie C, Du J, et al (2016) The association between telomere length and cancer risk in population studies. Sci Rep 6: 22243 10.1038/srep22243 26915412 PMC4768100

[25] Ridout KK, Ridout SJ, Price LH, Sen S, Tyrka AR (2016) Depression and telomere length: A meta-analysis. J Affect Disord 191: 237–247. 10.1016/j.jad.2015.11.052 26688493 PMC4760624

[26] Rode L, Nordestgaard BG, Bojesen SE (2015) Peripheral blood leukocyte telomere length and mortality among 64,637 individuals from the general population. J Natl Cancer Inst 107: djv074 10.1093/jnci/djv074 25862531

[27] Needham BL, Rehkopf D, Adler N, Gregorich S, Lin J, Blackburn EH, et al (2015) Leukocyte telomere length and mortality in the National Health and Nutrition Examination Survey, 1999–2002. Epidemiology 26: 528–535. 10.1097/EDE.0000000000000299 26039272 PMC4679150

[28] Haycock PC, Burgess S, Nounu A, Zheng J, Okoli GN, Bowden J, et al (2017) Association Between Telomere Length and Risk of Cancer and Non-Neoplastic Diseases: A Mendelian Randomization Study. JAMA Oncol 3: 636–651. 10.1001/jamaoncol.2016.5945 28241208 PMC5638008

[29] Charlson ME, Pompei P, Ales KL, MacKenzie CR (1987) A new method of classifying prognostic comorbidity in longitudinal studies: development and validation. J Chronic Dis 40: 373–383. 3558716 10.1016/0021-9681(87)90171-8

[30] Curtin LR, Mohadjer LK, Dohrmann SM, Montaquila JM, Kruszan-Moran D, Mirel LB, et al (2012) The National Health and Nutrition Examination Survey: Sample Design, 1999–2006. Vital Health Stat 2: 1–39. 22788053

[31] Johnson CL, Paulose-Ram R, Ogden CL, Carroll MD, Kruszon-Moran D, Dohrmann SM, et al (2013) National health and nutrition examination survey: analytic guidelines, 1999–2010. Vital Health Stat 2: 1–24. 25090154

[32] Cawthon RM (2002) Telomere measurement by quantitative PCR. Nucleic Acids Res 30: e47 10.1093/nar/30.10.e47 12000852 PMC115301

[33] Needham BL, Adler N, Gregorich S, Rehkopf D, Lin J, Blackburn EH, et al (2013) Socioeconomic status, health behavior, and leukocyte telomere length in the National Health and Nutrition Examination Survey, 1999–2002. Soc Sci Med 85: 1–8. 10.1016/j.socscimed.2013.02.023 23540359 PMC3666871

[34] Chen X, Redline S, Shields AE, Williams DR, Williams MA (2014) Associations of allostatic load with sleep apnea, insomnia, short sleep duration, and other sleep disturbances: findings from the National Health and Nutrition Examination Survey 2005 to 2008. Ann Epidemiol 24: 612–619. 10.1016/j.annepidem.2014.05.014 24985316 PMC4188508

[35] Parente V, Hale L, Palermo T (2013) Association between breast cancer and allostatic load by race: National Health and Nutrition Examination Survey 1999–2008. Psychooncology 22: 621–628. 10.1002/pon.3044 22290849

[36] Rehkopf DH, Needham BL, Lin J, Blackburn EH, Zota AR, Wojcicki JM, et al (2016) Leukocyte Telomere Length in Relation to 17 Biomarkers of Cardiovascular Disease Risk: A Cross-Sectional Study of US Adults. PLoS Med 13: e1002188 10.1371/journal.pmed.1002188 27898678 PMC5127504

[37] McKinlay SM (1994) Issues in design, measurement, and analysis for menopause research. Exp Gerontol 29: 479–493. 7925766 10.1016/0531-5565(94)90029-9

[38] Broer L, Amin N, Codd V, Oostra BA, Samani NJ, van Duijn CM. (2014) Telomere length variation reduces with age: evidence of survivor effect. Telomere and Telomerase 1.

[39] Gray KE, Schiff MA, Fitzpatrick AL, Kimura M, Aviv A, Starr JR. (2014) Leukocyte telomere length and age at menopause. Epidemiology 25: 139–146. 10.1097/EDE.0000000000000017 24213145 PMC3926311

[40] Jasienska G (2001) Why energy expenditure causes reproductive suppression in women: an evolutionary and bioenergetic perspective Reproductive ecology and human evolution New York: Aldine de Gruyter: 59–84.

[41] Bayne S, Li H, Jones ME, Pinto AR, van Sinderen M, Drummond A, et al (2011) Estrogen deficiency reversibly induces telomere shortening in mouse granulosa cells and ovarian aging in vivo. Protein Cell 2: 333–346. 10.1007/s13238-011-1033-2 21574023 PMC4875204

[42] Cha Y, Kwon SJ, Seol W, Park KS (2008) Estrogen receptor-alpha mediates the effects of estradiol on telomerase activity in human mesenchymal stem cells. Mol Cells 26: 454–458. 18719354

[43] Misiti S, Nanni S, Fontemaggi G, Cong YS, Wen J, Hirte HW, et al (2000) Induction of hTERT expression and telomerase activity by estrogens in human ovary epithelium cells. Mol Cell Biol 20: 3764–3771. 10.1128/mcb.20.11.3764-3771.2000 10805720 PMC85692

[44] Lee DC, Im JA, Kim JH, Lee HR, Shim JY (2005) Effect of long-term hormone therapy on telomere length in postmenopausal women. Yonsei Med J 46: 471–479. 10.3349/ymj.2005.46.4.471 16127770 PMC2815830

[45] Lin J, Kroenke CH, Epel E, Kenna HA, Wolkowitz OM, Blackburn E, et al (2011) Greater endogenous estrogen exposure is associated with longer telomeres in postmenopausal women at risk for cognitive decline. Brain Res 1379: 224–231. 10.1016/j.brainres.2010.10.033 20965155 PMC3057451

[46] O'Donovan A, Lin J, Tillie J, Dhabhar FS, Wolkowitz OM, Blackburn E, et al (2009) Pessimism correlates with leukocyte telomere shortness and elevated interleukin-6 in post-menopausal women. Brain Behav Immun 23: 446–449. 10.1016/j.bbi.2008.11.006 19111922 PMC2719778

[47] Passos JF, Saretzki G, von Zglinicki T (2007) DNA damage in telomeres and mitochondria during cellular senescence: is there a connection? Nucleic Acids Res 35: 7505–7513. 10.1093/nar/gkm893 17986462 PMC2190715

[48] von Zglinicki T, Serra V, Lorenz M, Saretzki G, Lenzen-Grossimlighaus R, Gessner R, et al (2000) Short telomeres in patients with vascular dementia: an indicator of low antioxidative capacity and a possible risk factor? Lab Invest 80: 1739–1747. 11092534 10.1038/labinvest.3780184

[49] Serra V, von Zglinicki T, Lorenz M, Saretzki G (2003) Extracellular superoxide dismutase is a major antioxidant in human fibroblasts and slows telomere shortening. J Biol Chem 278: 6824–6830. 10.1074/jbc.M207939200 12475988

[50] Aviv A (2002) Telomeres, sex, reactive oxygen species, and human cardiovascular aging. J Mol Med (Berl) 80: 689–695. 12436345 10.1007/s00109-002-0377-8

[51] Vasa M, Breitschopf K, Zeiher AM, Dimmeler S (2000) Nitric oxide activates telomerase and delays endothelial cell senescence. Circ Res 87: 540–542. 10.1161/01.res.87.7.540 11009557

[52] Stice JP, Lee JS, Pechenino AS, Knowlton AA (2009) Estrogen, aging and the cardiovascular system. Future Cardiol 5: 93–103. 10.2217/14796678.5.1.93 19371207 PMC3972065

[53] Xing D, Nozell S, Chen YF, Hage F, Oparil S (2009) Estrogen and mechanisms of vascular protection. Arterioscler Thromb Vasc Biol 29: 289–295. 10.1161/ATVBAHA.108.182279 19221203 PMC2700771

[54] Rufer N, Brummendorf TH, Kolvraa S, Bischoff C, Christensen K, Wadsworth L, et al (1999) Telomere fluorescence measurements in granulocytes and T lymphocyte subsets point to a high turnover of hematopoietic stem cells and memory T cells in early childhood. J Exp Med 190: 157–167. 10.1084/jem.190.2.157 10432279 PMC2195579

[55] Minamino T, Miyauchi H, Yoshida T, Ishida Y, Yoshida H, Komuro I. (2002) Endothelial cell senescence in human atherosclerosis: role of telomere in endothelial dysfunction. Circulation 105: 1541–1544. 10.1161/01.cir.0000013836.85741.17 11927518

[56] Rewak M, Buka S, Prescott J, De Vivo I, Loucks EB, Kawachi I, et al (2014) Race-related health disparities and biological aging: does rate of telomere shortening differ across blacks and whites? Biol Psychol 99: 92–99. 10.1016/j.biopsycho.2014.03.007 24686071 PMC4610356

[57] McCreary ML, Cunningham JN, Ingram KM, Fife JE (2006) Stress, culture, and racial socialization: Making an impact. Handbook of multicultural perspectives on stress and coping: 487–513.

[58] Yen YC, Lung FW (2013) Older adults with higher income or marriage have longer telomeres. Age Ageing 42: 234–239. 10.1093/ageing/afs122 22951603 PMC3575119

[59] Mainous AG 3rd, Everett CJ, Diaz VA, Baker R, Mangino M, Codd V, et al (2011) Leukocyte telomere length and marital status among middle-aged adults. Age Ageing 40: 73–78. 10.1093/ageing/afq118 20817935 PMC3000178

[60] Sbarra DA (2009) Marriage protects men from clinically meaningful elevations in C-reactive protein: results from the National Social Life, Health, and Aging Project (NSHAP). Psychosom Med 71: 828–835. 10.1097/PSY.0b013e3181b4c4f2 19661186 PMC3625249

[61] Engstrom G, Hedblad B, Rosvall M, Janzon L, Lindgarde F (2006) Occupation, marital status, and low-grade inflammation: mutual confounding or independent cardiovascular risk factors? Arterioscler Thromb Vasc Biol 26: 643–648. 10.1161/01.ATV.0000200100.14612.bb 16357315

[62] Scafato E, Galluzzo L, Gandin C, Ghirini S, Baldereschi M, Capurso A, et al (2008) Marital and cohabitation status as predictors of mortality: a 10-year follow-up of an Italian elderly cohort. Soc Sci Med 67: 1456–1464. 10.1016/j.socscimed.2008.06.026 18675500

[63] Molloy GJ, Stamatakis E, Randall G, Hamer M (2009) Marital status, gender and cardiovascular mortality: behavioural, psychological distress and metabolic explanations. Soc Sci Med 69: 223–228. 10.1016/j.socscimed.2009.05.010 19501442 PMC2852675

[64] Michael YL, Berkman LF, Colditz GA, Holmes MD, Kawachi I (2002) Social networks and health-related quality of life in breast cancer survivors: a prospective study. J Psychosom Res 52: 285–293. 12023125 10.1016/s0022-3999(01)00270-7

[65] Zhuravliova E, Barbakadze T, Zaalishvili E, Chipashvili M, Koshoridze N, Mikeladze D. (2009) Social isolation in rats inhibits oxidative metabolism, decreases the content of mitochondrial K-Ras and activates mitochondrial hexokinase. Behav Brain Res 205: 377–383. 10.1016/j.bbr.2009.07.009 19616040

