# Supplementary figures and images for: Decline in telomere length by age and effect modification by gender, allostatic load and comorbidities in National Health and Nutrition Examination Survey (1999-2002)
Source: PLoS One. 2019 Aug 30;14(8):e0221690. doi: 10.1371/journal.pone.0221690 (PMC6716670; doi:10.1371/journal.pone.0221690)

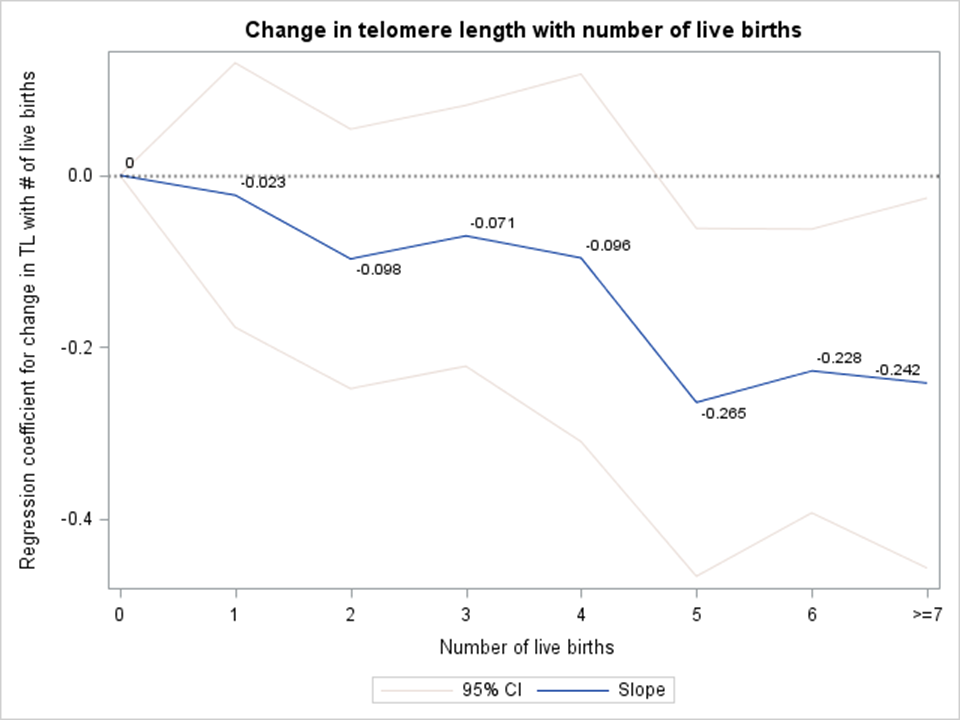

Supplement: S1 Fig — Y-axis represents the slope of change in telomere length with the number of live births. Regression coefficients adjusted for ethnicity, education, poverty income ratio, and body mass index. (TIF) [file pone.0221690.s001.tif]

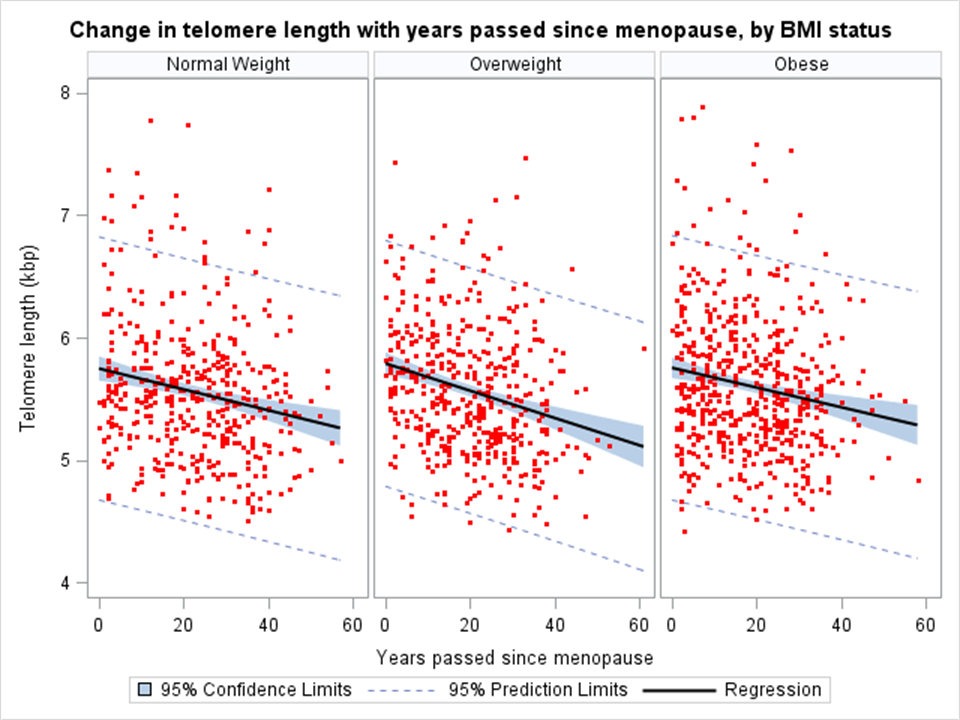

Supplement: S2 Fig — The slope for each weight category is: Normal weight (β = -0.0101; p = 0.001), Overweight (β = -0.0111; p = 0.005) and Obese (β = -0.0076; p = 0.007). Estimates adjusted for ethnicity, education, poverty income ratio, and body mass index. (TIF) [file pone.0221690.s002.tif]
