# Supplementary material for: Decline in telomere length by age and effect modification by gender, allostatic load and comorbidities in National Health and Nutrition Examination Survey (1999-2002)
Source: PLoS One. 2019 Aug 30;14(8):e0221690. doi: 10.1371/journal.pone.0221690 (PMC6716670; doi:10.1371/journal.pone.0221690)
Supplement: S1 Table — (DOCX) [file pone.0221690.s003.docx]

**S1 Table. Comorbid Conditions Included in Charlson’s Comorbidity Index**

| **SN** | **Condition** | **Definition of the condition** | **Weight** |
| --- | --- | --- | --- |
|  | Myocardial infarction | Participant’s self-report of ever being diagnosed with a heart attack or myocardial infarction. | 1 |
|  | Congestive heart failure | Participant’s self-report of ever being diagnosed with congestive heart failure. | 1 |
|  | Peripheral vascular disease | Ankle Brachial Blood Pressure Index of <0.90 in either leg [1]. | 1 |
|  | Cerebrovascular disease | Participant’s self-report of ever being diagnosed with stroke or cerebrovascular disease. | 1 |
|  | Dementia | Cognitive functioning was assessed by the Wechsler Adult Intelligence Scale (WAIS), third edition [2]. Dementia was defined as a score of more than 1.5 SD from mean WAIS or use of dementia medication (galantamine, rivastigmine, memantine, donepezil, or tacrine) [3]. | 1 |
|  | Pulmonary disease | Participant’s self-report of ever being diagnosed with asthma or chronic bronchitis or wheezing in the chest. | 1 |
|  | Connective tissue disorder | Connective tissue disorder was defined as participant’s self-report of ever being diagnosed with arthritis, or rheumatoid arthritis or osteoporosis; currently taking prescription antirheumatic drugs or glucocorticoid medications or prednisone or cortisone. | 1 |
|  | Liver disease | Participant’s self-report of ever being diagnosed with a liver condition. | 1 |
|  | Diabetes | Diabetes was defined as: a previous diagnosis of diabetes, or current use of diabetic pills or insulin, or a hemoglobin A1c level of 6.5% or greater, or a fasting plasma glucose level of ≥126 mg/dL, or 2-hour plasma glucose level of ≥200 mg/dL [4]. | 1 |
|  | Diabetes complications | Diabetes complications were considered if the participant reported retinopathy, unhealed ulcer/sore within four weeks, numbness in hands-feet, pain/tingling in hands feet and pain in either leg while walking due to diabetes. | 2 |
|  | Renal disease | Renal disease was defined by ever diagnosis of weak/failing kidneys or received dialysis in past 12 months, or a glomerular filtration rate (GFR)< 60 [5], or presence of microalbuminuria (urine albumin-to-creatinine ratio of ≥30 mg/g) [6]. GFR was estimated using the equation developed by Modification of Diet in Renal Disease Study Group [5]. | 2 |
|  | Cancer | Participant’s self-report of ever being diagnosed with cancer or malignancy of any type. | 2 |
|  | Leukemia | Participant’s self-report of ever being diagnosed with leukemia. | 2 |
|  | Lymphoma | Participant’s self-report of ever being diagnosed with lymphoma. | 2 |
|  | Moderate or severe liver disease | Participant’s self-report of ever being diagnosed with a liver condition. | 3 |
|  | HIV | Laboratory confirmed cases with HIV antibody. | 6 |

**References**

1. Selvin E, Erlinger TP: Prevalence of and risk factors for peripheral arterial disease in the United States: results from the National Health and Nutrition Examination Survey, 1999-2000. *Circulation* 2004, 110(6):738-743.
2. Johnson CL, Paulose-Ram R, Ogden CL, Carroll MD, Kruszon-Moran D, Dohrmann SM, Curtin LR: National health and nutrition examination survey: analytic guidelines, 1999-2010. *Vital Health Stat 2* 2013(161):1-24.
3. Hong CH, Falvey C, Harris TB, Simonsick EM, Satterfield S, Ferrucci L, Metti AL, Patel KV, Yaffe K: Anemia and risk of dementia in older adults: findings from the Health ABC study. *Neurology* 2013, 81(6):528-533.
4. Menke A, Casagrande S, Geiss L, Cowie CC: Prevalence of and Trends in Diabetes Among Adults in the United States, 1988-2012. *JAMA* 2015, 314(10):1021-1029.
5. Levey AS, Bosch JP, Lewis JB, Greene T, Rogers N, Roth D: A more accurate method to estimate glomerular filtration rate from serum creatinine: a new prediction equation. Modification of Diet in Renal Disease Study Group. *Ann Intern Med* 1999, 130(6):461-470.
6. Ricardo AC, Fischer MJ, Peck A, Turyk M, Lash JP: Depressive symptoms and chronic kidney disease: results from the National Health and Nutrition Examination Survey (NHANES) 2005-2006. *Int Urol Nephrol* 2010, 42(4):1063-1068.
