# Supplementary material for: Decline in telomere length by age and effect modification by gender, allostatic load and comorbidities in National Health and Nutrition Examination Survey (1999-2002)
Source: PLoS One. 2019 Aug 30;14(8):e0221690. doi: 10.1371/journal.pone.0221690 (PMC6716670; doi:10.1371/journal.pone.0221690)
Supplement: S2 Table — (DOCX) [file pone.0221690.s004.docx]

***S2 Table. Multivariable Regression for Factors Associated with Telomere Length- NHANES 1999-2002***

|  | **Adjusted Model ^a^** | | |
| --- | --- | --- | --- |
|  | **β** | **95%CI** | **p-value** |
| Age | **-0.0142** | **-0.0157, -0.0127** | **<0.001** |
| Gender (Reference= Male) |  |  |  |
| *Female* | 0.0238 | -0.0283, 0.076 | 0.3574 |
| Race/Ethnicity (Reference= NH White) |  |  |  |
| *Hispanic* | 0.0991 | -0.0379, 0.2361 | 0.1498 |
| *NH Black* | **0.1922** | **0.0972, 0.2873** | **0.0003** |
| *Other* | 0.0461 | -0.0958, 0.188 | 0.5116 |
| Educational Status (Reference=High School) |  |  |  |
| *<12th Grade* | **-0.1483** | **-0.2153, -0.0813** | **<0.001** |
| *College Graduate* | 0.0176 | -0.0483, 0.0834 | 0.5892 |
| Marital Status (Reference= Married/with Partner) |  |  |  |
| *Divorced/Widowed/Separated* | **-0.1284** | **-0.1915, -0.0653** | **0.0003** |
| *Never Married* | **0.2850** | **0.2084, 0.3617** | **<0.001** |
| Family PIR | -0.0237 | -0.0494, 0.002 | 0.069 |
| Biomarkers of allostatic load |  |  |  |
| *Systolic blood pressure* | **-0.0055** | **-0.0067, -0.0042** | **<0.001** |
| *Diastolic blood pressure* | -0.0008 | -0.0025, 0.0009 | 0.3465 |
| *Heart rate* | 0.0004 | -0.0012, 0.0019 | 0.6409 |
| *Total cholesterol* | **-0.0015** | **-0.0021, -0.0009** | **<0.001** |
| *High-density lipoprotein cholesterol* | 0.0005 | -0.0012, 0.0021 | 0.5603 |
| *BMI (kg/m2)* | **-0.0094** | **-0.0132, -0.0056** | **<0.001** |
| *Glycosylated hemoglobin* | **-0.0713** | **-0.0989, -0.0438** | **<0.001** |
| *C-reactive protein* | **-0.0816** | **-0.124, -0.0392** | **<0.001** |
| *Albumin* | -0.1308 | -0.9485, 0.687 | 0.7459 |
| Allostatic load (Reference=Low) |  |  |  |
| *High* | **-0.1626** | **-0.21, -0.1153** | **<0.001** |
| Charleston Comorbidity Index | **-0.0670** | **-0.077, -0.057** | **<0.001** |

DV: Telomere length in kbp; **^a^** Adjusted for gender, race/ethnicity, education, PIR, and physical activity. Abbreviations: BMI: body mass index, CI: confidence interval, NH: Non-Hispanic, PIR: poverty income ratio. p-value less than 0.05 are bold.
