# Supplementary material for: Decline in telomere length by age and effect modification by gender, allostatic load and comorbidities in National Health and Nutrition Examination Survey (1999-2002)
Source: PLoS One. 2019 Aug 30;14(8):e0221690. doi: 10.1371/journal.pone.0221690 (PMC6716670; doi:10.1371/journal.pone.0221690)
Supplement: S3 Table — (DOCX) [file pone.0221690.s005.docx]

***S3 Table. Multivariable Regression for Women’s Reproductive History Associated with Telomere Length- NHANES 1999-2002***

|  | **Adjusted Model ^a^** | | |
| --- | --- | --- | --- |
|  | **β** | **95%CI** | **p-value** |
| Parity (Reference= Nulliparous) |  |  |  |
| *One* | -0.0082 | -0.1467, 0.1303 | 0.904 |
| *Two* | -0.0118 | -0.1468, 0.1232 | 0.860 |
| *Three* | 0.0517 | -0.0972, 0.2006 | 0.483 |
| *Four* | 0.0912 | -0.1126, 0.295 | 0.368 |
| *Five* | -0.0351 | -0.2377, 0.1675 | 0.726 |
| *Six* | 0.0536 | -0.1098, 0.2171 | 0.507 |
| *Seven and more* | 0.0819 | -0.1363, 0.3001 | 0.449 |
| Menopausal status (Reference = No) |  |  |  |
| *Menopaused* | -0.0089 | -0.1008, 0.0829 | 0.8436 |
| Age at menopause | -0.0004 | -0.0057, 0.0048 | 0.8717 |
| Years passed since menopause | 0.0003 | -0.0051, 0.0057 | 0.9108 |

DV: Telomere length in kbp; **^a^** Adjusted for age, ethnicity, education, PIR, and body mass index. Abbreviations: CI: confidence interval, PIR: poverty income ratio. p-value less than 0.05 are bold.
